# Retrospective analysis of acute HBV infections occurred in 1978–79 and 1994–95 in North-East Italy: increasing prevalence of BCP/pre-core mutants in sub-genotype D3

**DOI:** 10.1186/s12879-019-4713-9

**Published:** 2020-01-28

**Authors:** Roberto Bruni, Umbertina Villano, Stefania Taffon, Michele Equestre, Elisabetta Madonna, Paola Chionne, Angela Candido, Stefano Dettori, Giulio Pisani, Maria Rapicetta, Flavia Bortolotti, Anna Rita Ciccaglione

**Affiliations:** 1 Department of Infectious Diseases, Istituto Superiore di Sanità, Rome, Italy; 2 Department of Neurosciences, Istituto Superiore di Sanità, Rome, Italy; 3 Notified Body 0373, Istituto Superiore di Sanità, Rome, Italy; 4 National Centre for the Control and Evaluation of Medicines, Istituto Superiore di Sanità, Rome, Italy; 5 Department of Experimental and Clinical Medicine, University of Padua, Padua, Italy

**Keywords:** Hepatitis B virus, HBV, Genotype, Sub-genotype, Basal core promoter, Pre-core, Mutant

## Abstract

**Background:**

At the end of the 1970s, in Italy more than 2% of the general population was HBsAg carrier. In the late ‘70s and late ‘80s, two remarkable events might have impacted on HBV strains transmitted in North-East Italy: (a) the increased HBV incidence due to parenteral drugs between 1978 and 1982; (b) the preventive anti-HIV educational campaign, started locally in 1985.

**Methods:**

To address if those events impacted on circulating HBV variants, acute cases occurred in North-East Italy in 1978–79 (*n* = 50) and 1994–95 (*n* = 30) were retrospectively analysed. HBV sequences obtained from serum samples were subjected to phylogenetic analysis and search for BCP/pre-core and S mutations.

**Results:**

HBV-D was the most prevalent genotype in both 1978–79 (43/50, 86%) and 1994–95 (24/30, 80.0%), with HBV-A in all but one remaining cases. Among HBV-D cases, sub-genotype HBV-D3 was the most prevalent (25/29, 86.2% in 1978–79; 13/16, 81.2% in 1994–95), with HBV-D1 and HBV-D2 in the remaining cases. All HBV-A cases were sub-genotype A2.

Single and multiple BCP/pre-core mutations, responsible for HBeAg(−) hepatitis, were detected in 6/50 (12%) cases in 1978/79 vs. 12/30 (40.0%) in 1994/95 (*p* = 0.006). They were found exclusively in HBV-D; in the most abundant sub-genotype, HBV-D3, they were detected in 2/25 (8%) cases in 1978–79 vs. 6/13 (46%) in 1994–95 (*p* = 0.011). No vaccine escape S mutations were observed. The IDU risk factor was significantly more frequent in 1994–95 (8/30, 26.7%) than in 1978–79 (4/50, 8%) (*p* = 0.048).

**Conclusions:**

The above mentioned epidemiological and public health events did not affect the proportion of genotypes and sub-genotypes that remained unchanged over 16 years. In contrast, the proportion of BCP/pre-core mutants increased more than three-fold, mostly in HBV-D3, a sub-genotype highly circulating in IDUs; drug abuse likely contributed to the spread of these mutants.

The findings contribute to explain a previously described major change in HBV epidemiology in Italy: the proportion of HBeAg(−) cases in the carrier cohort changed from low in late 1970s, to high at the beginning of the 2000s. In addition to other recognized factors, the increased circulation of BCP/pre-core mutants likely represents a further factor that contributed to this change.

## Background

Hepatitis B virus (HBV) infection is a global health problem concerning nearly 257 million chronic carriers at risk of cirrhosis and liver cancer [1]. HBV is an enveloped Hepadnavirus with a circular incomplete double stranded DNA genome of 3.2 Kb [2]. Its genetic variability is greater than that of any other DNA virus, as a result of viral genome replication by a reverse transcriptase lacking the proof-reading function [3].

Sequence analysis has shown that specific nucleotide variations play a role in pathogenesis and/or have impact in public health issues. Among them, mutations in the basal core promoter/pre-core (BCP/pre-core) region (associated with HBeAg negative hepatitis - a severe form of chronic liver disease - and with fulminant acute hepatitis) and those in the S coding region, especially in the main target of antibodies - the “a” determinant (associated with escape from vaccine induced immunity) are particularly well documented [4].

On the basis of nucleotide sequence analysis, HBV has been so far classified in ten genotypes (A to J) and several sub-genotypes, based on divergences of ≥7.5% and ≥ 4%, respectively [5, 6].

HBV genotypes have an ethno-geographic distribution, genotypes D (HBV-D) and A (HBV-A) being the most ubiquitous and responsible for the majority of infections in Northern and Southern Europe [7]. Very high degree of heterogeneity has been described for genotype D, with ten sub-genotypes (D1 to D10) so far reported [8, 9]. The divergences are strictly associated to population-related factors such as spread rate of epidemics and main routes of transmission. During the 1940s–1950s (that included the 2nd World War), HBV-D divergence and epidemic exponential growth took place as a result of unsafe medical practices and unscreened blood and blood derivatives. Another epidemic, mainly linked to HBV-D3, took place in the 1960s–1970s and involved subjects with high risk behaviour, such as intravenous drug users (IDU) [10]. HBV genotype A (particularly sub-genotype A2) largely spread between 1960s and early 1980s as a result of sexual transmission [11].

In Italy, the thirty years between 1940s and 1970s were crucial for the spread of HBV epidemics. At the end of the 1970s, more than 2% of the general population was HBsAg carrier, though with geographical differences; in those years, the circulation of sub-genotypes and viral mutants with clinical relevance were poorly studied because of the absence of appropriate routinely applied laboratory methods. In the following 20–30 years, HBV epidemiology significantly changed as a result of the improvements in socio-economic conditions and the implementation of compulsory vaccination, that started in Italy in 1991 [12].

The study of the HBV strains circulating in a geographical area is usually carried out by studying viruses from chronic patients. However, in most chronic carriers it is unknown when HBV infection was acquired: the transmission event might date back as few as weeks/months or as much as decades. In contrast, the study of acute cases provides an accurate overview of the strains actually transmitted over the enrolment period: being 6 months the upper limit for incubation time, it can be safely assumed that transmission did not occur earlier than 6 months before onset of symptoms.

In the ‘80s and ‘90s, two remarkable events might have impacted on HBV strains transmitted in North-East Italy: (a) a significant increase of acute HBV infections in young males and a parallel increased proportion of drug addicts were observed between 1978 and 1982 [13]; (b) national preventive measures linked to the anti-HIV educational campaign started to be implemented, in 1985 in the area object of the present study, in 1988 at national level. However, whether or not those events produced changes in the HBV strains circulating in this Italian area is unknown. The only available data about HBV strains circulating in North and North-East Italy were from chronic carriers (rather than acute cases) enrolled several years later, in 1993–2005 and 2000–2004 [11, 14]; conversely, studies from acute cases in Italy were either carried out in Southern Italy or, as part of a multicentre study, only included a small survey from North Italy. None of them included acute cases that had occurred earlier than 1999; thus, no data about HBV strains circulating in acute infections in ‘80s and early ‘90s are available [15–17].

The aim of the present study was to evaluate if the epidemics observed in 1978–1982 in North East Italy as well as the anti-HIV educational campaigns (started locally in 1985 and nationally in 1988) had any impact on HBV genotypes/sub-genotypes and clinically relevant mutants circulating in acute cases in this Italian area. The aim was addressed by comparing HBV sequences from acute cases enrolled in 1978–79 and 1994–95, i.e. before and after those events. Two viral genome regions of HBV (a 374 nt BCP/pre-core/core region and a 1295 nt region spanning the entire S open reading frame) were sequenced and subjected to phylogenetic analysis as well as search for BCP/pre-core and S mutations.

## Methods

### Patients and sera

Details on the design and the results of the “historical” survey were previously published [13, 18]. A prospective study started in Padua (North-East Italy) in 1978 at the Department of Infectious Diseases acting as a referral centre for the whole urban area and villages of the peripheral area (550,000 inhabitants). All consecutive patients with a diagnosis of acute hepatitis B, based on serum detection of HBsAg and anti-HBc IgM and a ALT level equal to or greater than five-fold the normal during the acute phase of the illness, were enrolled. All the patients were followed-up for possible evolution of acute infection into chronic: only 1.1% of 183 acute cases became chronic HBsAg carriers [19]. In the present study, available stored sera of consecutive patients, demonstrated to have cleared HBsAg, from two different periods, 1978–79 and 1994–95, were evaluated for HBV genotype/sub-genotype as well as for clinically relevant mutations by sequencing the BCP/pre-core and S viral genome regions. No patients had received lamivudine or steroid treatment during the acute phase of illness. Written informed consent was obtained from each patient participating in the study. Information on demographic characteristics, risk factors for HBV infection and clinical course were obtained from patient records.

Serum samples were collected at clinical presentation of the disease and tested for HBV markers (HBsAg, anti-HBc IgM, HBeAg, anti-HBe, HBV-DNA), anti-HDV and anti-HCV by commercial ELISA and in-house assays as previously described [19].

### Extraction of HBV-DNA and sequencing of BCP/pre-core and S gene regions

Viral DNA was extracted from 200 μL serum using the EZ1 Virus Mini Kit v.2.0 (Qiagen, Hilden, Germany) following the manufacturer’s instructions. HBV-DNA was amplified by polymerase chain reaction (PCR) with the Platinum Taq DNAPolymerase (Invitrogens, Life Technologies Corporation, Monza, Italy) as previously described [20]. The PCR products were purified using the Amicon® Ultra-0.5 Centrifugal Filter Devices in accordance with the manufacturer’s instructions. Sequencing reactions were performed using the Genome Lab DTCS Quick Start Kit (Beckman Coulter, Inc., Fullerton, CA) and were run on an automated DNA sequencer (Beckman Coulter, Inc.). Raw output sequences were analysed by the Chromas software and the BioEdit Package. A 374 bp sequence (encompassing BCP/pre-core and the 5′ portion of the core coding region) and a 1295 bp sequence (encompassing the entire coding region of the S gene, including pre-S1, pre-S2 and S) were finally obtained and analysed.

### Phylogenetic analysis and search for BCP/pre-core and S mutations

Representative full genome reference sequences of all known HBV genotypes (A, B, C, D, E, F, G, H, I) and sub-genotypes of genotype D (D1 to D10) and A (A1 to A7) were downloaded from the NCBI database (https://www.ncbi.nlm.nih.gov/nucleotide/). Their proper 374 nt region (encompassing BCP/pre-core and the 5′ portion of the core coding region) and 1295 nt region (encompassing the entire coding region of the S gene) were aligned with sequences to be genotyped or sub-genotyped by ClustalW in Bioedit.

The following reference sequences were used for genotyping: AF297623 and AB076678 (genotype A); D00329 and D23679 (genotype B); D16665 and AY057947 (genotype C); FJ904402 and FJ904395 (genotype D); AB091255 and X75664 (genotype E); AY090459 and AY090461 (genotype F); AB056513 and AB056514 (genotype G); AY090454 and AY090460 (genotype H); FJ023663 and EU835242 (genotype I); AB486012 (genotype J).

For sub-genotyping of genotype D sequences, preliminary phylogenetic trees with multiple representative sequences for each sub-genotype showed all investigated sequences were D1 or D2 or D3. To reduce as much as possible the total number of sequences in the final phylogenetic tree, three reference each for sub-genotypes D1, D2 and D3 and one reference each for the remaining sub-genotypes (D4 to D10) were included in the dataset. The following references were used: FJ904402, JN642167, AJ344116 (sub-genotype D1); Z35716, X97849, AB078033 (sub-genotipe D2); AY233291, EU594434, X65257 (sub-genotype D3); AB048701 (sub-genotype D4); DQ315779 (sub-genotype D5); KF170740 (sub-genotype D6); FJ904395 (sub-genotype D7); FN594769 (sub-genotype D8); JN664919 (sub-genotype D9); KX357625 (sub-genotype D10).

Genotype A sequences were sub-genotyped using the following references: JN182318 (sub-genotype A1); HE576989 (sub-genotype A2); AB194951 (sub-genotype A3); AY934764 (sub-genotype A4); FJ692613 (sub-genotype A5); GQ331047 (sub-genotype A6); FN545833 (sub-genotype A7).

The best nucleotide substitution model for phylogenetic analysis was estimated for each dataset by the Models tool in MEGA6 [21]. Then, phylogenetic trees were constructed using the Maximum Likelihood approach in MEGA6 with the best substitution model. The reliability of phylogenetic trees was tested by bootstrap analysis (1000 replicates). To check for robustness of results, additional trees were also built by a completely different approach, Maximum Parsimony, that assumes no substitution model.

Search for known BCP/pre-core and S mutations was carried out by Bioedit analysis of aligned sequences; to search for amino acid differences, the nucleotide sequences were translated in the appropriate open reading frame.

### Statistical analysis

Comparison of categorical data was carried out by Fisher’s exact test, continuous data by T Student’s. A *p* < 0.05 was considered to be significant.

## Results

The demographic, clinical and virological characteristics at hospital admission of the 80 acute hepatitis B patients included in the study are reported in Table 1. All patients were HBsAg and anti-HBc IgM positive. They were enrolled in two periods 16 years apart: 50 cases in 1978–79 and 30 cases in 1994–95. The two groups were similar for demography, distribution of HBV markers and clinical features.

**Table 1:** Demographic, clinical and virological characteristics at hospital admission of 80 HBsAg/anti-HBc IgM positive patients in 1978–79 and 1994–95 surveys of acute hepatitis B in North-East Italy

Years	1978–79	1994–95
Number of patients	50	30
Sex M/F (index)	28/22 (1.3)	18/13 (1.4)
Median age (range)	32 (23–85)	25 (15–78)
HBeAg positive (%)	34 (68.0)	17 (56.7)
HBeAg negative (%)	16 (32.0)	13 (43.3)
Anti-HBe positive (%)	29 (58.0)	17 (56.7)
Mean Log_10_ HBV DNA (range)	5.51 (3.29–8.51)	4.34 (1.48–8.71)
Anti-HDV positive (%)	2 (4.0)	3 (10.0)
Anti-HCV positive (%)	12 (24.0)	8 (25.8)
Mean hospital days (range)	37.6 (18–77)	19.5 (8–26)
Mean IU ALT (range)	2280 (850–3760)	2678 (658–4600)
Risk exposure:		
IDU (%)	4/50 (8.0) ^§^	8/30 (26.7) ^§^
other parenteral (sexual, household contact) (%)	20/50 (40.0)	7/30 (23.3)
unknown (%)	26/50 (52.0)	16/30 (53.3)
HBV genotype (%):		
A	6/50 (12.0)	6/30 (20.0)
D	43/50 (86.0)	24/30 (80.0)
E	1/50 (2.0)	–
HBV-D sub-genotype (%) ^a^		
D1	1/29 (3.4)	1/16 (6.2)
D2	3/29 (10.3)	2/16 (12.5)
D3	25/29 (86.2)	13/16 (81.2)

^§^ *p* = 0.048, Fisher’s test

^a^sub-genotype could be determined for 29 genotype D cases in the 1978–79 group and 16 cases in the 1994–95 group

Co-infection with HDV and HCV was detected in 4 and 24% cases, respectively, in the 1978–79 group vs. 10 and 25.8% in the 1994–95 group (*p* = 0.36 for HDV co-infection and *p* = 0.79 for HCV co-infection).

In one half cases, none of the investigated risk factors for HBV infection was reported. The IDU risk factor was significantly more frequent in the 1994–95 group (8/30, 26.7%) than in the 1978–79 group (4/50, 8%) (*p* = 0.048). The frequency of other risk factors (sexual or household contacts and professional exposure) did not differ between the two groups (*p* = 0.16).

### Genotyping/subgenotyping

**Fig. 1:**
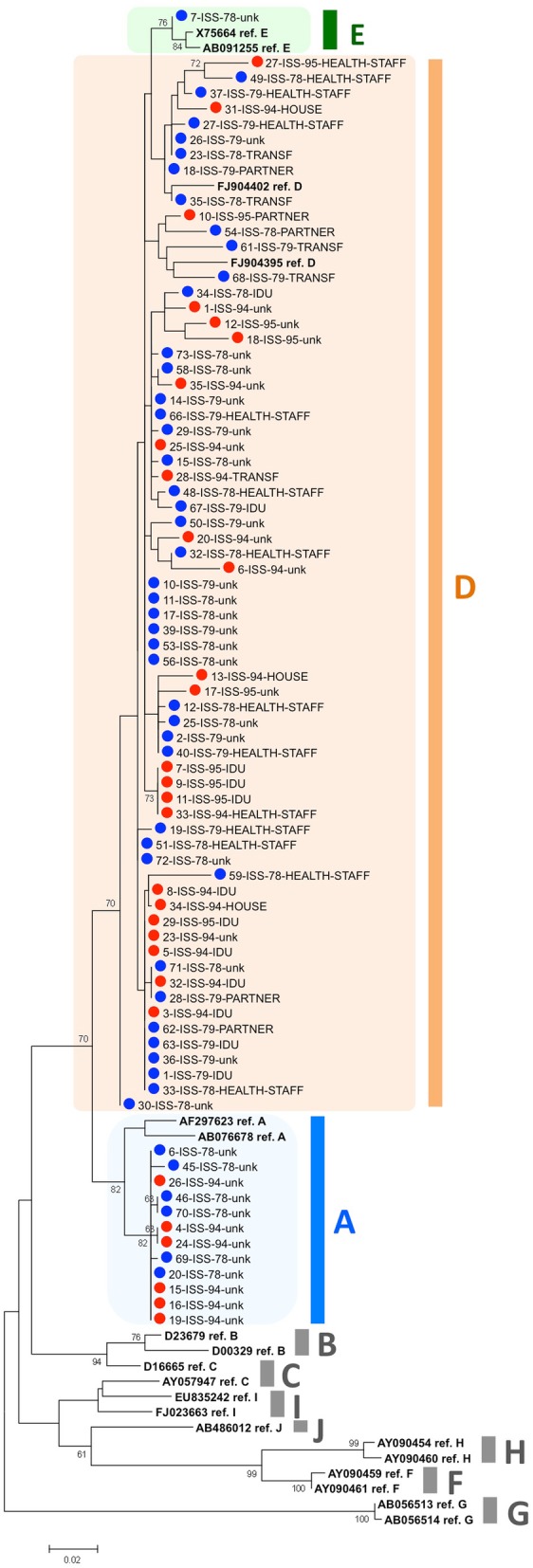
Genotyping by phylogenetic analysis of BCP/pre-core/core gene sequences from 80 patients. The sequence dataset included 19 reference sequences. The phylogenetic tree was constructed by the ML approach with the K2 + G + I substitution model (preliminary estimated to be the best substitution model for the dataset under analysis by the Models tool in MEGA6). Bootstrap values > 70 are reported. A blue circle marks sequences from 1978 to 79, a red circle those from 1994 to 95; reference sequences are shown in bold

Figure 1 shows genotyping of HBV from 80 patients by phylogenetic analysis of BCP/pre-core/core region (374 nt). Genotype D largely predominates in both groups (43/50, 86% in the 1978/79 group and 24/30, 80.0% in the 1994/95 group); genotype A was detected in the remaining cases (6/50, 12% in 1978/79 and 6/30, 20.0%% in 1994/95) with one only exception, a genotype E virus in the 1978/79 group (1/50, 2%).

**Fig. 2:**
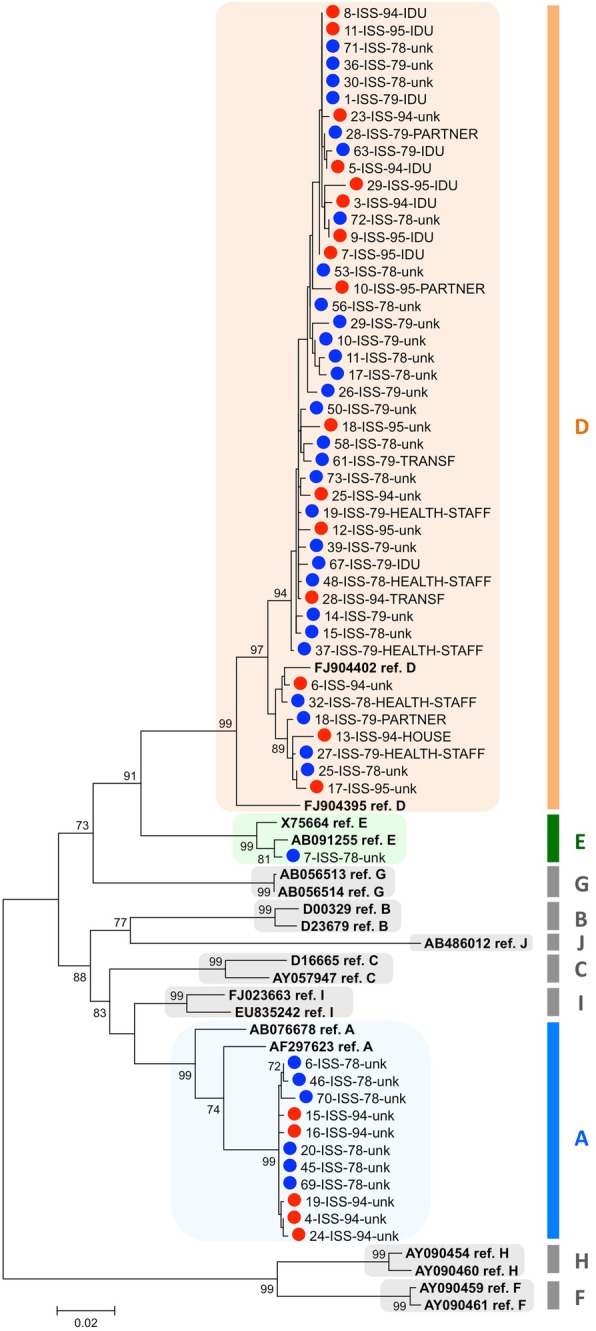
Genotyping by phylogenetic analysis of S gene sequences from 57 patients. The sequenced region overlapped the Pre-S1, Pre-S2 and S regions of the S open reading frame. The sequence dataset included 19 reference sequences. The phylogenetic tree was constructed by the ML approach with the GTR + G + I substitution model (preliminary estimated to be the best substitution model for the dataset under analysis by the Models tool in MEGA6). Bootstrap values > 70 are reported. A blue circle marks sequences from 1978 to 79, a red circle those from 1994 to 95; reference sequences are shown in bold

Figure 2 shows HBV genotyping by phylogenetic analysis of the entire S region, including pre-S1, pre-S2 and S (1295 nt); due to limited sample amount, sequencing of the S region could be carried out in 57 of the 80 samples. The genotype assignment by S analysis was consistent with the genotype assignment by BCP/pre-core/core analysis in all patients. The genotype distribution in this subset of 57 cases was: 45 genotype D, 11 genotype A, 1 genotype E.

The D and A sequences from the S region were subjected to further phylogenetic analysis to determine the sub-genotype, as previously reported by other authors [22].

**Fig. 3:**
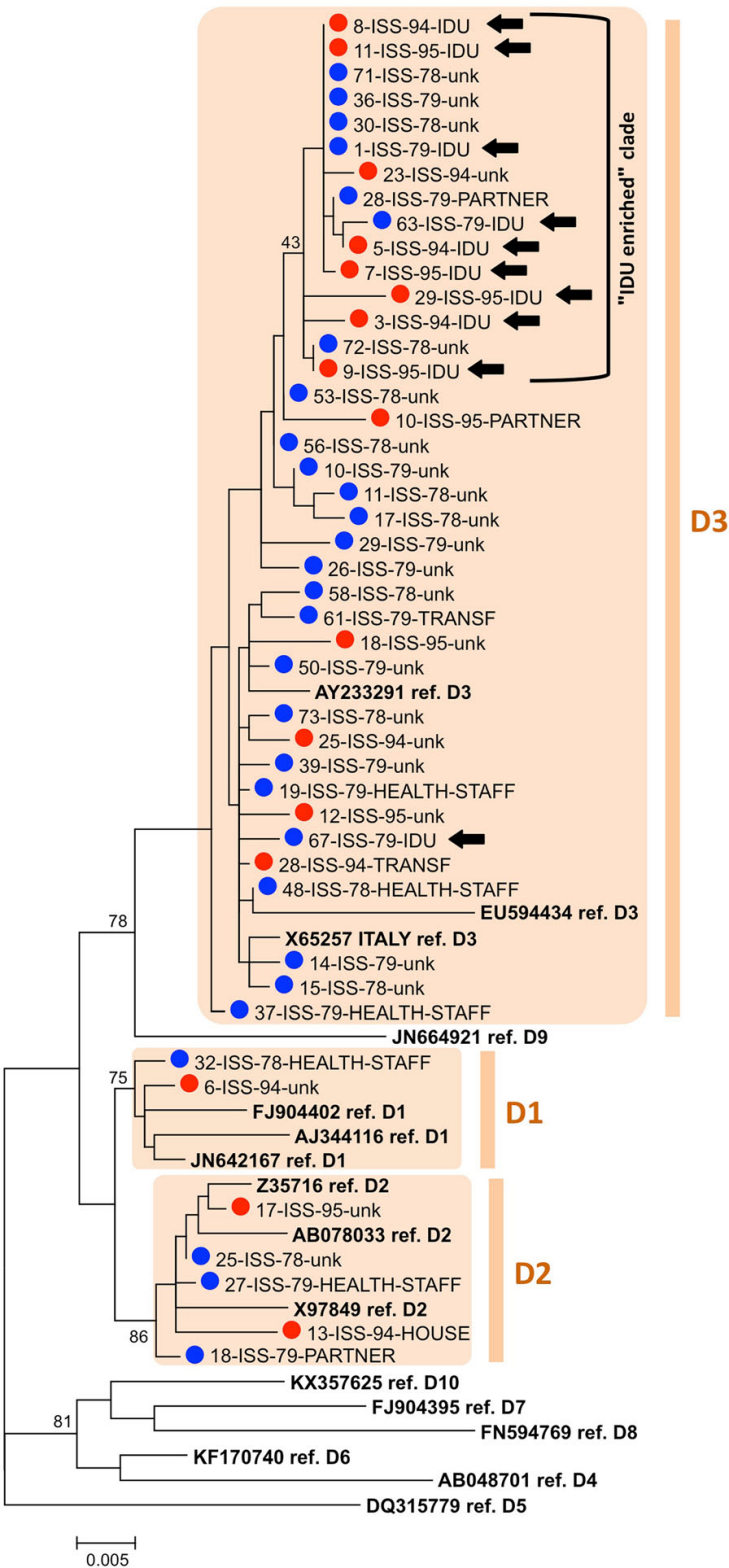
Subtyping by phylogenetic analysis of the 45 genotype D sequences from S region. The sequence dataset included subtype D1 to D10 reference sequences. The phylogenetic tree was constructed by the ML approach with the GTR + G + I substitution model (preliminary estimated to be the best substitution model for the dataset under analysis by the Models tool in MEGA6). A blue circle marks sequences from 1978 to 79, a red circle those from 1994 to 95; reference sequences are shown in bold. The tree is drawn to scale, with branch lengths measured in the number of substitutions per site. A black arrow marks sequences whose patient reported the IDU risk factor. Bootstrap values > 70 are reported (exception: the bootstrap value at the node of the branch comprising most sequences from cases reporting the IDU risk factor)

Figure 3 reports the subtyping results for HBV-D (29 sequences from 1978 to 79 and 16 from 1994 to 95). HBV-D3 was the most prevalent in both groups (25/29, 86.2% in 1978–79, 13/16, 81.2% in 1994–95); HBV-D1 and HBV-D2 were observed in the remaining cases (HBV-D1: 1 case in 1978/79, 1 case in 1994/95; HBV-D2: 3 cases in 1978/79 and 2 cases in 1994/95).

All but one sequences from IDUs clustered in a separate branch of the D3 subtype, together with some other sequences, but the bootstrap value did not reach significance (Fig. 3, “IDU enriched” clade); phylogenetic analysis was repeated by a completely different approach, Maximum Parsimony, in which the phylogenetic tree that requires the smallest total number of evolutionary events to explain the observed sequence data is identified: sequences from IDUs clustered in a statistically supported clade, larger than in the previous tree (Additional file 1: Figure S1). Overall, these results suggest the variants observed in IDUs are more genetically related to each other than to most remaining D3 sequences, likely a consequence of restricted circulation/transmission in this population group both in 1978–79 and in 1994–95; however, different phylogenetic approaches show different ability to highlight their relatedness.

Subgenotyping of HBV-A (11 S sequences: 6 from 1978 to 79 and 5 from 1994 to 95) showed all of them were HBV-A2 (Additional file 2: Figure S2).

### Search for BCP/pre-core and S mutations

Table 2 reports the patterns of viral mutations detected in the BCP/pre-core region. The known clinically relevant T1753C, A1762T, G1764A, G1896A and G1899A mutations were detected as both single and multiple mutations (up to five simultaneous mutations per strains were observed).

**Table 2:** Patterns of BCP/pre-core mutants in two groups of acute hepatitis B patients from North-East Italy occurred 16 years apart

	1978–79			1994-95		
	Total	HBeAg(-)	HBV-D3	Total	HBeAg(-)	HBV-D3
Number of patients	50	16	25^a^	30	13	13^a^
BCP/pre-core mutation pattern (n° of patients):						
G1764A				4	1	3
G1896A	3	1	1	2	2	1
T1753C/A, G1896A				1	1	
A1762T, G1764A	1			1		
G1896A, G1899A	1		1	1	1	
T1753C/A, A1762T, G1764A, G1896A				1	1	1
T1753C/A, A1762T, G1764A, G1899A	1	1				
T1753C/A, A1762T, G1764A, G1896A, G1899A				2	2	1
Patients with at least one BCP/pre-core mutation (n°,%)	6 (12.0%)^#^	2 (12.5%)^§^	2 (8.0%)^^^	12 (40.0%)^#^	8 (61.5%)^§^	6 (46.0%)^^^

^a^due to sample volume limitations, only a subset of HBV-D cases could be sub-genotyped by analysis of S region: 29/43 from the 1978–79 group and 16/24 from the 1994–95 group. Sub-genotype D3 was by far the most abundant in both groups, with 25 and 13 cases, respectively

^#^ *p* = 0.006 (Fisher’s exact test); all cases with BCP/pre-core mutations were HBV-D

^§^ *p* = 0.016 (Fisher’s exact test)

^^^ *p* = 0.011 (Fisher’s exact test)

The prevalence of isolates with at least a single mutation was 6/50 (12%) in the 1978/79 group vs. 12/30 (40.0%) in the 1994/95 group (*p* = 0.006).

All mutations were detected exclusively in genotype D sequences; the prevalence in HBV-D was 6/43 (14.0%) in the 1978/79 group vs. 12/24 (50.0%) in the 1994/95 group (*p* = 0.003).

Among the 45 sub-typed HBV-D cases, sub-genotype D3 was the most abundant (25 and 13 cases in the 1978–79 and the 1994–95 groups, respectively) and allowed for sub-genotype specific analysis. As reported in Table 2, BCP/pre-core mutations in HBV-D3 sequences were detected in 2/25 (8%) cases in the 1978–79 group vs. 6/13 (46%) cases in the 1994–95 group (*p* = 0.011).

Analysis of the subset of HBeAg(−) cases showed BCP/pre-core mutants were 2/16 (12.5%) in the 1978/79 vs. 8/13 (61.5%) in 1994/95 (*p* = 0.016). In this latter group, 7 out of the 8 mutant strains harboured (alone or in combination with other BCP/pre-core mutations) the G1896A mutation, well known to determine a STOP codon responsible for premature termination of the HBeAg precursor synthesis.

Analysis of the predicted 226 amino acid sequences from the S region did not show any previously described vaccine escape mutations (data not shown).

## Discussion

In North-East Italy, remarkable changes of the epidemiology of acute hepatitis B were observed between 1978 and 1982; they were mainly related to the spread of parenteral drug abuse; the epidemics peaked in 1982–1983 [13]. However, possible changes of the circulating HBV strains remained uninvestigated, mainly because the routine methods for molecular characterisation became largely applied only several years later.

The first descriptions of the HBV strains circulating in Northern Italy were reported in 2006 and 2007, when two studies described the strains detected in two surveys of chronic carriers enrolled, respectively, between 2000 and 2004 and between 1993 and 2005 [11, 14]. In the present retrospective study, HBV strains from the same area (North-East Italy) dating back 1978–79 and 1994–95 were analysed, but exclusively from acute cases: this provides an accurate overview of the strains actually transmitted in those years because, in contrast with chronic carriers, the time point of infection can be safely defined.

The studied acute cases had preceded (1978–1979 cases) or had occurred in the early years of (1994–95 cases) public health interventions that will then prove crucial for the modification of HBV epidemiology in Italy: the wide application of the preventive measures linked to the anti-HIV educational campaign (started in 1988) and the implementation of HBV mass vaccination (started in 1991).

The results show that the same genotypes and sub-genotypes were stably transmitted in this Italian area through 16 years. In particular, HBV-D was the most prevalent in both the 1978–79 and the 1994–95 groups, while HBV-A accounted for the remaining cases, with the unique exception of an HBV-E strain detected in one single patient in 1978. Even at the sub-genotype level, the D3, D2 and D1 in decreasing abundance and the A2 were detected in both groups at similar frequency. Thus, the epidemiological and public health events occurred between the two investigated periods did not impact on the distribution of transmitted HBV genotypes/subgenotypes.

Search for BCP/pre-core mutations showed no such mutations in genotype A cases. At least for position 1896, the finding is in agreement with previous reports: the G1896A pre-core mutation has been rarely observed in genotype A, as it would disrupt an essential stem-loop structure in the ε signal essential for pre-genomic RNA packaging [23]. In contrast, the analysis has highlighted a significantly increased prevalence of BCP/pre-core mutants in acute HBV-D infections, particularly in HBV-D3, from 1978 to 79 to 1994–95.

These findings can help to explain a previously observed major change in epidemiology of HBV in Italy over 30 years. By the end of the 1970s, in Italy most chronic carriers were HBeAg(+): the carrier cohort showed low proportion of HBeAg(−) subjects; however, at the beginning of the year 2000 the scenario was inverted, with high proportion of HBeAg(−) carriers [12, 24, 25]. Two factors had been invoked to explain this change: (a) reduction of the overall HBV incidence (as result of prevention campaigns and vaccination) that reduced replenishment of the carrier cohort with new HBeAg(+) carriers and (b) aging of the HBV carrier cohort, leading to selection of BCP/pre-core mutants and, consequently, change from HBeAg(+) to HBeAg(−) phenotype [12, 24, 25]. The present study suggests a third cooperating factor: the increased transmission of BCP/pre-core mutants. The increased detection of BCP/pre-core mutants in acute cases implied replenishment of the carrier cohort with an increased proportion of HBeAg(−) cases; in turn, the widespread parenteral drug abuse likely resulted in increased likelihood to transmit BCP/pre-core mutant viruses, due to their preferential circulation in IDUs, giving a contribution to their spread. The epidemic of acute HBV infections observed in North East Italy between 1978 and 1982 was mainly caused by parenteral drug abuse: the proportion of IDUs reached 42% of total acute cases [13]. In the next years, between 1983 and 1988, the HBV incidence dropped and the proportion of IDUs in acute cases also decreased markedly; however, the number of new subjects starting drug abuse did not decline and the proportion of IDUs, after a minimum in 1987, increased again in 1988 [13]. In the present study, the proportion of IDUs in the 1994–95 group was 26.7%, so 3-fold more frequent than in 1978–79 group and also higher than in 1988; significantly, the IDU risk factor was mostly reported by patients infected by HBV-D3, a sub-genotype highly circulating among IDUs [8]. The tendency of D3 variants found in IDUs to group into a phylogenetic clade (Fig. 3 and Additional file 1: Figure S1, “IDU enriched” clade) suggests that their circulation was limited, to a certain degree, to IDUs; however, spread into the general population had also occurred, because strains from cases with different or unknown risk factors were also interspersed in the same clade. Thus, increased circulation of BCP/pre-core mutants in acute cases likely cooperated with the two other previously reported factors (see above) to change the proportion of HBeAg(−) cases in the Italian carrier cohort, from the low proportion at the end of 1970s to the high proportion observed at the beginning of the 2000s [12, 24, 25].

Regarding analysis of the S region, an above mentioned study of HBV in the same geographical area in chronic carriers between 1993 and 2005 reported frequent mutation of the “a” determinant, including the G145A/R mutation involved in vaccine escape [11].

In the present work no such mutations were observed. Two main differences between the two studies are likely responsible for this result: (a) different infection status (acute vs chronic cases) and (b) different sampling period (1978–79 and 1994–95 vs 1993–2005). It is not surprising that no escape mutations were observed in the present study. Acute cases had been enrolled either several years before (1978–79 group) or just a few years after (1994–95 group) the introduction of mandatory vaccination, which started in Italy in 1991 in two population cohorts: 3 months of age newborns and 12 years old children. Thus, in 1978–79 any strain with a vaccine escape polymorphism would have not been subjected to any vaccine selection, while in 1994–95 the vaccinated cohorts were 0–4 years and 12–16 years, so the selection pressure for escape mutants was likely still negligible. In contrast, the survey analysed in De Maddalena et al. included chronic cases, sampled about a decade later (between 1993 and 2005): the reported escape mutants could have been either the result of natural selection during chronic infection because of their ability to evade the host immune response or had been directly acquired when escape mutant strains had possibly started spreading (cases were in fact sampled up to 14 years after introduction of mandatory vaccination) [11].

## Conclusions

In conclusion, the present study shows that the proportion of genotypes and sub-genotypes in acute cases in North-East Italy remained unchanged over 16 years, from 1978 to 79 to 1994–95. In contrast, the proportion of BCP/pre-core mutants increased more than three-fold, mostly in HBV-D3, a sub-genotype highly circulating in IDUs; accordingly, the IDU risk factor was also more frequently reported, suggesting that IDUs contributed to the spread of the BCP/pre-core mutants.

These findings contribute to explain a previously described major change in HBV epidemiology in Italy: the proportion of HBeAg(−) cases in the carrier cohort changed from a low proportion in late 1970s to a high proportion at the beginning of the 2000s. In addition to other recognized factors, the increased circulation of BCP/pre-core mutants likely represents a further factor that contributed to this change.

## Supplementary information

**Additional file 1:** **Figure S1.** Subtyping by phylogenetic analysis of the 45 genotype D sequences from S region by Maximum Parsimony approach. The sequence dataset included the same subtype D1 to D10 reference sequences reported in Fig. 3. The phylogenetic tree was constructed by the maximum Parsimony approach. A blue circle marks sequences from 1978 to 79, a red circle those from 1994 to 95; reference sequences are shown in bold. A black arrow marks sequences whose patient reported the IDU risk factor. Bootstrap values > 60 are shown.

**Additional file 2:** **Figure S2.** Subtyping by phylogenetic analysis of the 11 genotype A sequences from S region. The sequence dataset included subtype A1 to A7 reference sequences. The phylogenetic tree was constructed by the ML approach with the K2 + G substitution model (preliminary estimated to be the best substitution model for the dataset under analysis by the Models tool in MEGA6). A blue circle marks sequences from 1978 to 79, a red circle those from 1994 to 95; reference sequences are shown in bold. The tree is drawn to scale, with branch lengths measured in the number of substitutions per site.

## Data Availability

BCP/pre-core/core and S gene sequences of the present study were deposited in GenBank. The accession numbers are as follows: MN702627 to MN702706 and MN702707 to MN702763. If any trouble is had accessing the data, the data will be available from the corresponding author upon request.

## Abbreviations

BCP/pre-core: Basal Core Promoter/pre-core
HBV: Hepatitis B virus;
IDU: Intravenous drug user
