# Supplementary figures and images for: Retrospective analysis of acute HBV infections occurred in 1978–79 and 1994–95 in North-East Italy: increasing prevalence of BCP/pre-core mutants in sub-genotype D3
Source: BMC Infect Dis. 2020 Jan 28;20:78. doi: 10.1186/s12879-019-4713-9 (PMC6988336; doi:10.1186/s12879-019-4713-9)

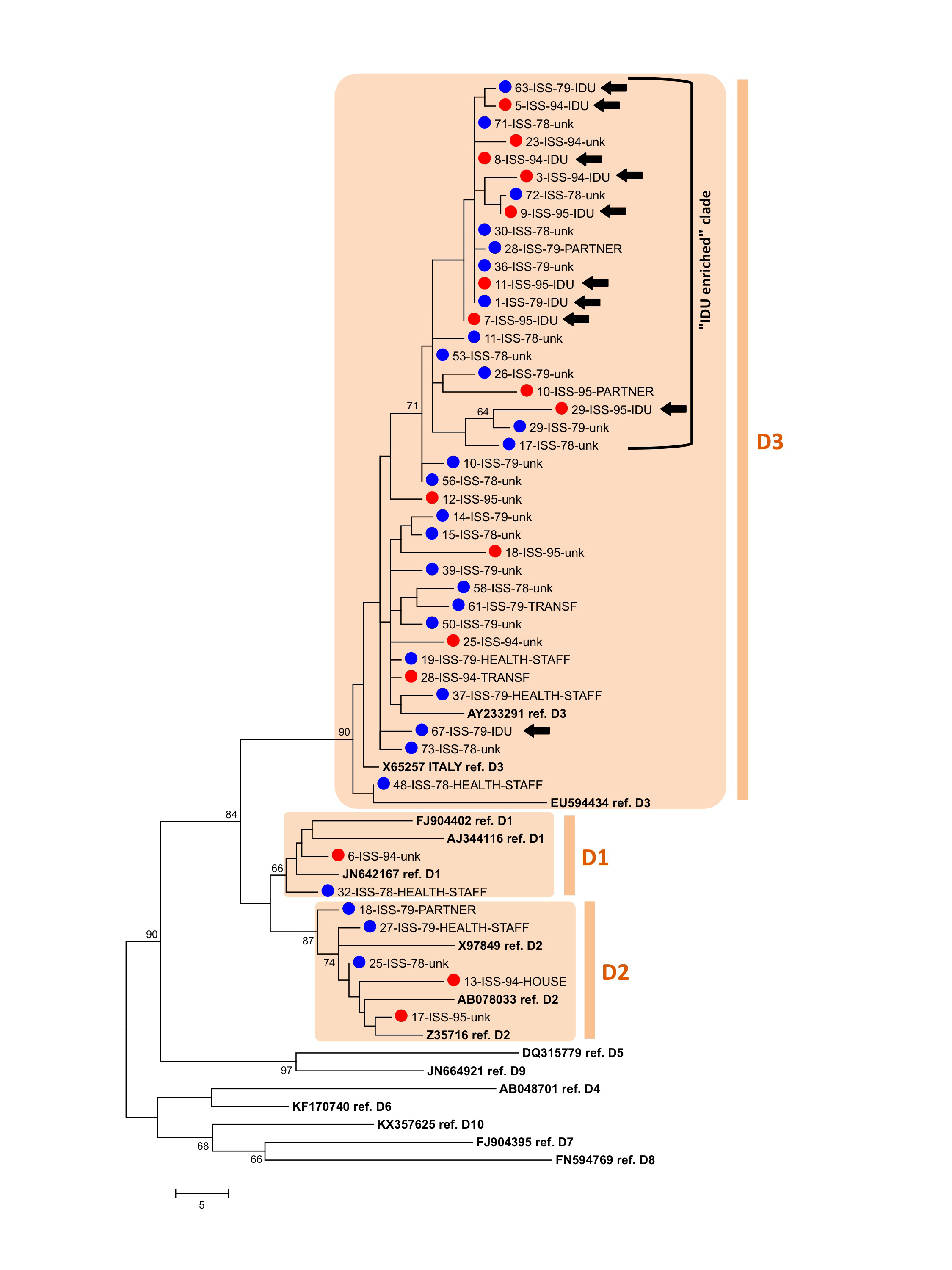

Supplement: Supplementary file 1 — Additional file 1: Figure S1. Subtyping by phylogenetic analysis of the 45 genotype D sequences from S region by Maximum Parsimony approach. The sequence dataset included the same subtype D1 to D10 reference sequences reported in Fig. 3. The phylogenetic tree was constructed by the maximum Parsimony approach. A blue circle marks sequences from 1978 to 79, a red circle those from 1994 to 95; reference sequences are shown in bold. A black arrow marks sequences whose patient reported the IDU risk factor. Bootstrap values > 60 are shown. [file 12879_2019_4713_MOESM1_ESM.tif]

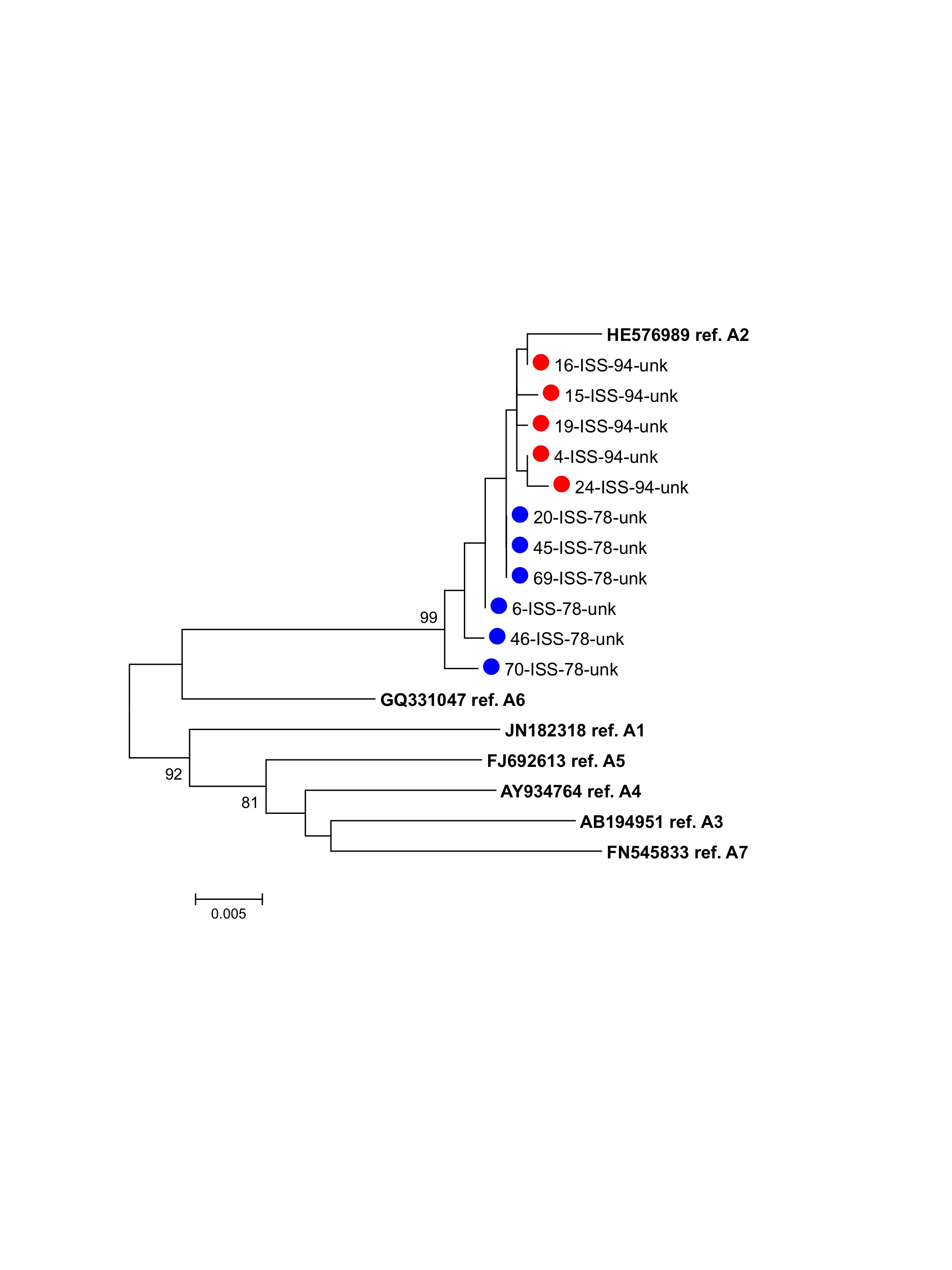

Supplement: Supplementary file 2 — Additional file 2: Figure S2. Subtyping by phylogenetic analysis of the 11 genotype A sequences from S region. The sequence dataset included subtype A1 to A7 reference sequences. The phylogenetic tree was constructed by the ML approach with the K2 + G substitution model (preliminary estimated to be the best substitution model for the dataset under analysis by the Models tool in MEGA6). A blue circle marks sequences from 1978 to 79, a red circle those from 1994 to 95; reference sequences are shown in bold. The tree is drawn to scale, with branch lengths measured in the number of substitutions per site. [file 12879_2019_4713_MOESM2_ESM.tif]
